# West Nile Virus Lineage 2 Strain in Greece, 2012

**DOI:** 10.3201/eid1905.121418

**Published:** 2013-05

**Authors:** Serafeim C. Chaintoutis, Alexandra Chaskopoulou, Taxiarchis Chassalevris, Philip G. Koehler, Maria Papanastassopoulou, Chrysostomos I. Dovas

**Affiliations:** Aristotle University of Thessaloniki, Thessaloniki, Greece (S.C. Chaintoutis, T. Chassalevris, M. Papanastassopoulou, C.I. Dovas),; University of Florida, Gainesville, Florida, USA (A. Chaskopoulou, P.G. Koehler);; US Department of Agriculture, Thessaloniki (A. Chaskopoulou)

**Keywords:** WNV, Greece, 2012 epidemic, sentinel chickens, molecular identification, viruses, West Nile virus, lineage 2 strain

**To the Editor:** West Nile virus (WNV) has been in Europe at least since the 1960s (*1*). Before 2010, WNV epidemics in Europe were caused mainly by lineage 1 strains. However, in 2010, a major WNV epidemic in Central Macedonia, Greece, was caused by a lineage 2 strain (Nea Santa-Greece-2010) (*2*). 

This strain also circulated during 2011 (*3*), causing a second epidemic among humans throughout the country (*4*). Although the virus was closely related to the goshawk-Hungary-2004 strain circulating in Hungary, Austria, and Italy (*5*–*7*), severe epidemics occurred only in Greece; 273 cases of West Nile neuroinvasive disease (WNND) in humans were reported during the 2 seasons (*2*,*4*).

A third epidemic occurred in 2012, and 109 WNND cases were reported (*8*). Until mid-August, most cases were in central (Attica; 29 cases) and northeastern Greece (East Macedonia and Thrace; 10 cases). In contrast, during the same period, only 3 cases were confirmed at the location of the 2010 epidemic epicenter, in Central Macedonia (*9*). This situation led to the question of whether the Nea Santa-Greece-2010 strain was responsible for the third epidemic in Greece.

In May 2012, for the second consecutive year, 12 sentinel chicken flocks (72 chickens) and 62 dry ice–baited (source of carbon dioxide) CDC mosquito traps (John W. Hock, Gainesville, FL, USA) were set in areas of Central Macedonia where WNV transmission had been high. The 3 objectives were to 1) monitor WNV activity by testing weekly for antibodies against WNV in sentinel chickens, 2) molecularly characterize the virus, and 3) assess population dynamics of the major vector species.

Serum from the sentinel chickens was tested for WNV-specific antibodies by using the ID-Screen West Nile Competition ELISA kit (IDvet, Montpellier, France). Seropositive chickens were removed from the sentinel flocks and replaced with seronegative chickens. 

No enzootic activity was detected until mid-August. Antibodies were first found on August 21, in 2 chickens from the rice-growing region of western Thessaloniki (Delta and Chalkidona municipalities). The seroconversion rate in these municipalities peaked on August 27, when 4 chickens were seropositive, and remained high until September 4. Seroconversion was detected for 10 more birds in the same and other bordering municipalities, all near the rice-growing region and flood plain of the Axios, Loudias, and Aliakmonas Rivers. WNV-specific antibodies were detected in 5 more chickens sampled on September 11, in 2 chickens sampled on September 18, and in 3 chickens sampled on September 25, bringing the total number of seroconverted chickens to 26 (26.5%) of 98 (original 72 + 26 replacements). The highest rates of seroconversion among chickens were detected near rice-growing areas, where *Culex* mosquito activity (mostly *Cx. pipiens*, followed by *Cx*. *modestus*) was also highest. *Culex* mosquito populations peaked in mid-July, ≈30 days before the first case in a human was detected, and maintained high activity until late September.

For the 26 chickens that seroconverted, RNA was retrospectively extracted from serum by using the QIAamp Viral RNA Mini Kit (QIAGEN, Hilden, Germany). Extracts were examined by using a 1-tube, real-time, reverse transcription PCR protocol and primers (WNPolUp, WNPolDo2) (*3*) and TaqMan probe (WNPolProb2: 5′-FAM-TCTCTCTCTTTCCCATCATGTTGT-BHQ1–3′) specific to the nonstructural (NS) 5 gene. WNV RNA was detected in 2 seropositive chickens from 2 locations, in samples taken about 1 week before seroconversion.

The positive RNA samples were reverse transcribed, and 3 overlapping fragments of the NS3 gene were amplified by PCR and then sequenced. Both complete NS3 sequences of 1,857 bp were identical (GenBank accession no. JX843471). Highest nucleotide sequence identity (99.8%) was to the strain Nea Santa-Greece-2010. Only 3 synonymous nucleotide substitutions consisting of transitions were identified, indicating minimum evolution of the virus during 2010–2012 (Figure). Molecular characterization of the 2 isolates actively circulating where cases in humans had been confirmed suggests that the strain responsible for the 2012 epidemic in Greece was again Nea Santa-Greece-2010.

**Figure:**
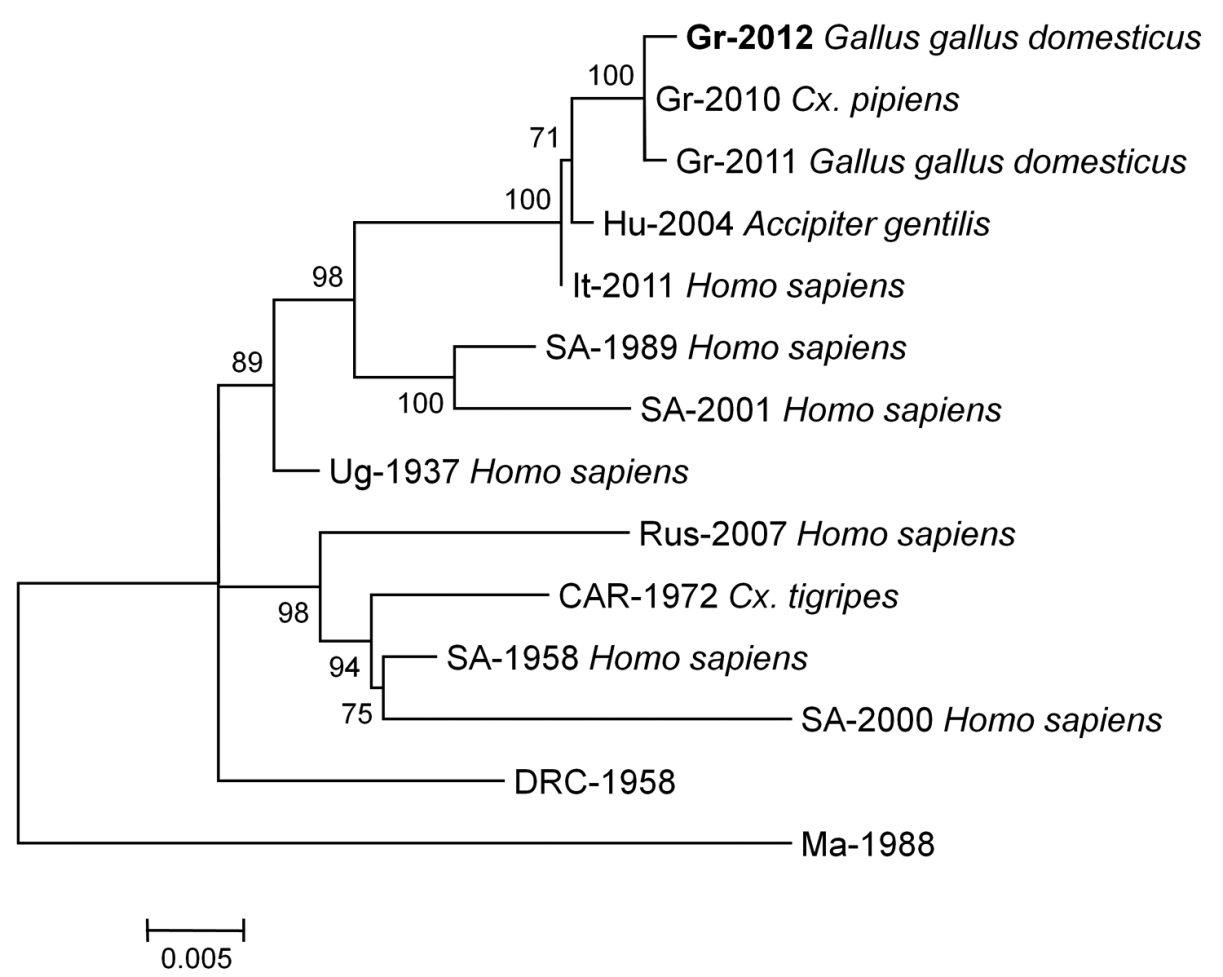
Phylogenetic tree inferred with maximum-likelihood analysis, based on complete nonstructural (NS) 3 nt sequences (1,863 bp) of lineage 2 West Nile virus strains. Isolation source is indicated in **boldface**. The general time reversible model with gamma distributed rates across sites and a fraction of sites assumed to be invariable (GTR + I + Γ) was selected as the best fitting nucleotide substitution model for the sequence dataset. The tree was mid-point rooted, and the numbers indicated on the branches are nonparametric bootstrap probabilities. Strain abbreviations indicate country, year, and GenBank accession number. Gr-2012: Greece, 2012, JX843471; Gr-2010: Greece, 2010, HQ537483; Gr-2011: Greece, 2011, JN398476; Hu-2004: Hungary, 2004, DQ116961; It-2011: Italy, 2011, JN858070; SA-1989: South Africa, 1989, EF429197; SA-2001: South Africa, 2001, EF429198; Ug-1937: Uganda, 1937, AY532665; Rus-2007: Russia, 2007, FJ425721; CAR-1972: Central African Republic, 1972, DQ318020; SA-1958a: South Africa, 1958, EF429200; SA-2000: South Africa, 2000, EF429199; DRC-1958: Democratic Republic of the Congo, 1958, HM147824; Mad-1988: Madagascar, 1988, HM147823. Scale bar indicates nucleotide substitutions per position. *Cx., Culex*.

The surveillance system successfully identified areas with increased levels of vector and WNV activity. This information was quickly disseminated to public health authorities so they could intensify control measures in the affected areas. After the first seroconversions in chickens were detected, 9 new WNND cases and 2 cases without central nervous system manifestations in humans were reported from residences near (6–15 km) the chicken coops that housed the seropositive birds (*8*). Of the 9 cases of WNND, 7 were reported after the rate of chicken seroconversion peaked.

The continuous occurrence of WNV epidemics in Greece indicates that the virus will probably remain a serious threat. This probability is further supported by the epidemic pattern in the United States, where ≈10 years after its introduction WNV is still causing large epidemics (*10*). Surveillance programs that can accurately determine public health risk and lead to timely vector control interventions are needed to prevent human infection. 

Particularly in areas such as Europe, where numerous strains of different virulence coexist, molecular identification of the circulating viruses is necessary for risk assessment. Captive sentinel chicken surveillance with repetitive sampling might be an informative tool. 
